# Investigating the Role of Normative Support in Atheists’ Perceptions of Meaning Following Reminders of Death

**DOI:** 10.3389/fpsyg.2022.913508

**Published:** 2022-07-05

**Authors:** Melissa Soenke, Kenneth E. Vail, Jeff Greenberg

**Affiliations:** ^1^Department of Psychology, California State University Channel Islands, Camarillo, CA, United States; ^2^Department of Psychology, Cleveland State University, Cleveland, OH, United States; ^3^Department of Psychology, University of Arizona, Tucson, AZ, United States

**Keywords:** terror management theory, atheism, mortality salience, normative support, meaning in life

## Abstract

According to terror management theory, humans rely on meaningful and permanence-promising cultural worldviews, like religion, to manage mortality concerns. Prior research indicates that, compared to religious individuals, atheists experience lower levels of meaning in life following reminders of death. The present study investigated whether reminders of death would change atheists’ meaning in life after exposure to normative support for atheism. Atheists (*N* = 222) were either reminded of death or a control topic (dental pain) and exposed to information portraying atheism as either common or rare, and then asked to rate their perceived meaning in life. Results showed that reminders of death reduced meaning in life among atheists who were told that atheism is common. Results were consistent with the view that atheism reflects the rejection of religious faith rather than a meaningful secular terror managing worldview. Discussion considers implications for maintaining healthy existential wellbeing, identifies limitations, and highlights future research directions.

## Introduction

While research into the psychological construct of meaning in life has grown tremendously in recent years, researchers lament the lack of work on sources of meaning (Schnell, 2011). According to terror management theory (TMT; Greenberg et al., 1986), humans’ cultural worldviews provide a meaningful sense of longevity in the face of mortality concerns. Religion is one widely researched worldview that TMT research has shown to be protective against concerns about death (see Soenke et al., 2013 for a review). It offers this protection through literal immortality beliefs, supernatural belief in an immortal soul that will live on after death. Religion is also often associated with meaning in life (see Hood et al., 2018 for a review). Conversely, a growing body of research indicates that atheists, individuals who do not believe in any supernatural agents or a soul, experience lower levels of meaning in life than religious individuals (Horning et al., 2011; Schnell and Keenan, 2011), particularly following reminders of death (Vail and Soenke, 2018). The present study further investigated that pattern, exploring whether death awareness would continue to undermine atheists’ meaning in life after exposure to normative support for atheism itself.

### Terror Management Theory, Religiosity, and Meaning in Life

Terror Management Theory (Greenberg et al., 1986) is an existential theory of human behavior based on the work of cultural anthropologist Becker (1971, 1973, 1975), that states that humans’ unique cognitive abilities allow for a sophisticated and abstract awareness that sets us apart as the only animal able to understand the scope and severity of our own mortality. The theory and associated research indicate that humans have developed two basic psychological constructs to manage the potential anxiety or “terror” that this understanding poses. First, cultural worldviews are sets of beliefs, standards, and values which imbue the world with order, meaning, and purpose and offer some form of either symbolic or literal permanence. Cultural worldviews can take many varying forms, but are all characterized by their role in providing a structure through which adherents can make sense of the world. These worldviews include prescriptions for achieving a sense of longevity. They can do this in two ways, either literally, as with belief in an immortal soul that will live on after death, or symbolically through cultural works that leave a lasting legacy by which we are remembered. Centrality components of worldviews include national identity, political orientation, and religious affiliation. Second, self-esteem is the sense that one is living up to the standards and values put forward by our cultural worldviews, and thus is a valuable contributor to a meaningful world. According to TMT, people manage their potential anxiety regarding their mortality by maintaining faith in these two constructs, and considerable energy is directed toward pursuing these goals.

Terror management theory’s *mortality salience hypothesis* proposes that if certain psychological structures (e.g., one’s cultural worldviews) help to protect individuals from concerns about death, then increasing mortality salience (MS) should strengthen people’s need for these systems (Rosenblatt et al., 1989). Considerable TMT research has found that reminders of mortality can lead people to seek out and maintain a sense of meaning by affirming their cultural worldviews (see Pyszczynski et al., 2015 for review). For example, when reminded of death (vs. control topic), American undergraduates prefer a pro-American essay over an essay that is critical of America (Greenberg et al., 1990). Similarly, university students majoring in natural sciences favored an evolutionary theory account of human origin over intelligent design following reminders of death (Tracey et al., 2011).

Research into the TMT dual process model of defense (Pyszczynski et al., 1999) demonstrates that distinct types of defenses are used when death is in conscious awareness and once it has been pushed out of conscious awareness but is easily accessible. Proximal defenses are used when death is in conscious focus and tend to be rational, threat focused, and designed to address the problem of death directly through denying vulnerability or distracting oneself (Pyszczynski et al., 1999). For example, denying vulnerability to health risk factors (Greenberg et al., 2000), increasing intentions to behave in healthy ways (Arndt et al., 2003; Taubman Ben-Ari and Findler, 2005), and distracting oneself with positive emotions (DeWall and Baumeister, 2007). Once thoughts of death are outside of conscious awareness, distal defenses of worldview defense and self-esteem maintenance and enhancement take over. Unlike proximal defenses, distal defenses (often called terror management defenses) aren’t so directly related to death, but instead bolster belief that one is a being of enduring value in a meaningful world. Because the present research is focused on variables that may affect atheists’ distal defenses, following prior research (Greenberg et al., 1997; Kosloff et al., 2018), we use a delay and distraction task so that death is no longer in conscious focus prior to measuring our dependent variable.

According to TMT, both religious and secular worldviews address the problem of mortality by offering a template for leaving a meaningful legacy here on earth (symbolic immortality), but religious worldviews are unique in that they also offer literal immortality through supernatural belief in an immortal soul that will live on after death. Many studies find that religious belief is associated with lower levels of anxiety generally and anxiety about death specifically (see Soenke et al., 2013 for a review), and that religious belief is associated with a myriad of other positive outcomes, like meaning in life, health, longevity, and general well-being (see Hood et al., 2018 for a review). TMT research has shown that MS motivates increased religiosity and strengthened faith in supernatural concepts among religious individuals (Norenzayan and Hansen, 2006; Jong et al., 2012; Vail et al., 2012). Affirming faith in religious concepts, such as an afterlife or creation stories also reduces death related thoughts and worldview defensiveness following MS (Dechesne et al., 2003; Schimel et al., 2007). In addition to protecting against concerns about death, research on sources of meaning indicates that *religious* sources of meaning are particularly predictive of overall meaning in life (Emmons, 2005; Schnell, 2010, 2011), and religious individuals, even those lower in religiosity, tend to score higher than atheists on measures of meaning in life (Horning et al., 2011; Schnell and Keenan, 2011).

### Terror Management Theory, Atheism, and Meaning in Life

Atheists, however, do not believe in any given religious supernatural concepts. Some have compellingly argued that one’s atheism can be weak or strong (e.g., Flew, 1984; Martin, 1992). Weak atheism is when people passively lack affirmative belief in god; individuals can either be unaware that they don’t believe, or they can be aware of it and capable of self-reporting it. Strong atheism is when one does not merely passively lack faith, but actively rejects it (also see Dawkins, 2006). Weak or strong, atheism does *not* describe the *presence* of belief in any *secular* worldview—it does not describe whether one knows much about science, strives to be compassionate toward others, wants to be remembered for their prowess on the basketball court, or believes in such secular virtues as civic engagement, teaching, farming, or raising a family. Instead, “atheism” *only* describes the *absence* of faith in supernatural concepts—at least the passive lack of faith and at most the assertive rejection of it.

Indeed, TMT research on atheists indicates that although atheists and religious believers both implicitly activate supernatural concepts following reminders of death (e.g., Jong et al., 2012), believers also express increased acceptance of faith in religious/supernatural concepts whereas atheists do not (Norenzayan and Hansen, 2006; Jong et al., 2012; Vail et al., 2012). Instead, MS can increase atheists’ explicit *rejection* of supernatural concepts (Jong et al., 2012, Study 1), and affirmations of natural (medical life extensions) but *not* supernatural (afterlife) concepts mitigate the effects of MS on their secular worldview defenses (Vail et al., 2018). Thus, at least among American atheists, the awareness of death may initially implicitly motivate the activation of supernatural concepts, but (at least under certain conditions) they can override and reject those implicit supernatural concepts and associated expressions of religious belief.

That rejection of, rather than acceptance of, a terror managing set of religious beliefs may leave atheists “groundless”—having rejected one permanence-promising system of meaning (religious concepts) without necessarily having affirmed another in its place (e.g., secular beliefs and values). That groundlessness may, at least temporarily, undermine atheists’ ability to maintain a sense of meaning in life when managing the increased awareness of death. To test that idea, Vail and Soenke (2018) recruited Christian and atheist participants, and either reminded them of death or a negative control topic (dental pain) using the classic MS prime consisting of two open ended questions (Rosenblatt et al., 1989). After a delay, participants completed an eight-item measure of their perceived meaning in life (Krause, 2007). Results indicated that Christians’ reported levels of meaning in life were not impacted by the MS or the control condition. However, whereas atheists in the control condition reported similar levels of meaning in life as the Christian participants, those in the MS condition showed a significant decrease in meaning in life. Thus, whereas religious participants were able to maintain a sense of meaning in life following a death reminder, atheists were vulnerable to reduced sense of meaning in life when reminded of mortality.

### Salient Prevalence of Atheism: Worldview Affirmation or Negation?

Much research has found religious belief to be associated with improved well-being and better ability to cope with negative life events, but some researchers question whether these benefits of religion are a result of the faith in religious content itself, or are better accounted for by the access to social support and validation that often come with religious belief (e.g., communities of fellow believers) but are less readily available to non-believers (Hood et al., 2018). Indeed, relationships between religion and wellbeing have been well accounted for by variables like perceived social support and social capital (Salsman et al., 2005; Stark and Maier, 2008; Yeary et al., 2012). Likewise, Horning et al. (2011) found that atheists showed lower levels of meaning in life and reported access to fewer sources of social support than their religious counterparts —even those low in religiosity.

Given that social support and validation appear to be a key ingredient in the link between religiosity and improved wellbeing, Galen (2015) argued that if atheists were to perceive similar social support and validation in their secular communities, they should experience similar psychological and health benefits. A potential illustration of this presumed effect can be seen by comparing data obtained in the United States and Norway. When asked, in 2018, if they believed in God, a minority (just 13%) of Americans said “no” (Hyrnowski, 2018) whereas a majority (52.9%) of Norwegians said “no” (European Values Survey, 2017). In line with Galen’s argument, research in the predominantly religious United States indicates religious engagement is positively associated with aspects of wellbeing like life satisfaction and happiness, whereas research in the predominantly secular Norway showed no significant differences between religious and non-religious individuals’ aspects of social support nor wellbeing (Kvande et al., 2015).

One important question is whether the prevalence of atheism itself plays a role in shoring up atheists’ wellbeing even in the face of existential threat. We identified two possible theoretical perspectives on the topic, leading to two competing hypotheses.

#### Worldview Affirmation Hypothesis

One perspective builds on two ideas. First: the assumption that the salient prevalence of atheism would somehow affirm atheists’ permanence-promising worldview beliefs, standards, or values. Second: the TMT idea that the perception of prevalent social support for one’s permanence-promising worldviews may help manage existential concerns about one’s impermanence.

Indeed, prior work has found that social support and consensus can play an important role in buffering against existential threat (for a review see Greenberg et al., 2014). When others reject one’s beliefs or hold a competing worldview, they raise the possibility that one’s own worldview might either be wrong or irrelevant. But when others share one’s worldview, that consensus helps affirm one’s worldview as a valid system of meaningful beliefs, standards, and values. For example, MS caused Germans to estimate greater social consensus for their political beliefs, and Americans to estimate greater consensus for their religious beliefs (Pyszczynski et al., 1996). Likewise, MS increased participants’ death-thought accessibility, but not after an affirmation of their self-worth and cultural values (Schmeichel and Martens, 2005; Vail et al., 2018).

It is notable that—at least in the United States—Christians make up over 70% of the population (Pew Research Center, 2015a), about 88% of the current (117th) and previous United States Congress is Christian (Pew Research Center, 2015b, 2021), and every single United States President has expressed belief in God (Pew Research Center, 2017). Christianity is pervasive and thoroughly integrated into the fabric of American society. In contrast, atheism in America does not enjoy the consensual validation of normative support and atheists are instead a relatively rare and often openly despised minority. Although the numbers of atheists range from 500 to 750 million worldwide (Zuckerman, 2007), anti-atheist prejudice in America is prevalent and strong (e.g., Edgell et al., 2006; Gervais et al., 2011; Jones, 2012) and most do not openly identify as atheists (Gervais and Najle, 2018). Compared to countless churches and faith-based groups, the largest American irreligious group—the Freedom from Religion Foundation—has just 32,000 members (∼0.000042% of the estimated number of atheists globally).

Such normative support for Christianity (but not atheism) could help explain why Christians (but not atheists) were able to maintain the perception of meaning in life even when reminded of death (Vail and Soenke, 2018). It might also suggest that if atheists perceived atheism to be prevalent (assuming prevalent atheism would somehow affirm atheists’ terror-managing beliefs in the same way prevalent Christianity affirms Christians’ terror-managing beliefs) they might be similarly protected. Thus, combining that assumption with these TMT ideas leads to the hypothesis that: among American atheists, MS should undermine perceived meaning in life (as in prior work, Vail and Soenke, 2018) when led to view religion as prevalent but not when led to view atheism as prevalent.

#### Worldview Negation Hypothesis

A competing view, however, recognizes—as in the definition of atheism, above—that atheism does not describe the *presence* of any particular secular worldview beliefs, standards, or values. Atheism is not, for example, humanist compassionate values, scientific understanding, or support for the New York Yankees; it doesn’t denote the adoption of particular theories of music, civics, or economic policies; and it isn’t a legacy from writing a book, having children of one’s own, or teaching the children of others. Atheism is completely mute about any such positive, affirmative investment in any terror-managing, permanence-promising secular worldview beliefs, standards, or values. Instead, atheism is merely the *absence* or even rejection of religious faith.

In line with that definition, this second view assumes that salient prevalence of atheism signals a negation of religious worldviews without necessarily affirming any particular terror-managing secular worldview in its place. That may create a “groundlessness” which may leave people open to reduced meaning, especially when aware of death. Nietzsche (1882) illustrated the problem in his famous “madman” passage, where an individual realizes with confidence that “God is dead” but laments that there is no clearly affirmed meaning system ready to take his place—the individual will have to take additional steps to create and affirm a coherent system of secular values. Frankl’s (1946) concept of the existential vacuum can, in this case, be interpreted in a similar vein: negating traditional worldviews without affirming and engaging a replacement worldview creates vulnerability to boredom, meaninglessness, and even crisis.

Thus, this view begins by assuming atheism denotes the absence of religious faith, rather than the presence of any given secular worldviews, and that salient prevalence of atheism would merely negate a widespread sociocultural system without necessarily affirming any particular secular terror-managing worldview in its place. Thus, this view predicts that: among American atheists, MS should undermine perceived meaning in life (as in prior work, Vail and Soenke, 2018) when led to view atheism as increasingly prevalent but not when led to view it as still rare.

### The Present Research

Whereas previous research has found that atheists suffered a reduction in the perception of meaning in life when reminded of death (Vail and Soenke, 2018), the present research explores that effect further. American atheists were first reminded of either death or a control topic (dental pain). They were then given information either emphasizing atheism as rare or as increasingly common (following Gervais, 2011). After these manipulations, they rated their sense of meaning in life (Krause, 2004). The worldview affirmation hypothesis assumes atheism somehow offers a permanence-promising set of secular beliefs, standards, and values, and thus predicted MS would undermine atheists’ meaning in life when they perceive atheism as rare but not when they perceive strong normative support for atheism. The worldview negation hypothesis, however, recognizes atheism entails the negation of religious terror-managing worldviews rather than the affirmation of any given secular terror-managing worldviews, and thus predicted MS would undermine atheists’ meaning in life when led to perceive strong normative support for atheism (negating religion but not affirming an alternative) rather than when led to perceive it as rare.

## Method

### Participants

A meta-analysis of prior MS manipulation research (Burke et al., 2010) found a large overall MS effect size of *r* = 0.35 (*d* = 0.75), though of course the true effect size may be smaller. Thus, using G*Power software (G*Power; Faul et al., 2007), we computed an *a priori* power analysis for an interaction (*F*-family tests, ANOVA interactions) for a minimum effect size threshold set to a medium size of *f* = 0.25, with power set to β = 0.80 for detecting the presence of such effects at α = 0.05, with one numerator *df* and four groups. This analysis recommended a minimum overall sample size of 128 participants. The obtained sample size (*N* = 222) met and exceeded that minimum recommendation, and a sensitivity power analysis showed the study was sensitive enough to detect effects as small as *f* = 0.18 (small-medium effect sizes). Sample size was determined before any data analysis.

In Fall 2018, 296 participants were recruited for participation by the research panel recruitment service Cloud Research using the religious screening item “*What religion or philosophy are you affiliated with, if any? (1) Christian; (2) Muslim;(3) Jewish; (4) Buddhist; (5) Hindu; (6) Spiritual (I believe supernatural beings do exist, but I do not follow a specific religion); (7) Agnostic (I’m not sure whether, or it is impossible to know whether, supernatural beings do or do not exist); (8) Atheist (I do not believe supernatural beings exist); and (9) Other _____.”* Panel members who selected “atheist” were eligible to participate. Of the 296 participants recruited, 32 participants failed to complete extensive portions of the study, 4 did not give consent to participate and one was 17 years old and so could not consent to participate. The religious screening item was administered again at the end of the study, to confirm that the respondents were indeed atheists, and an additional 37 participants identified themselves as having religious/spiritual beliefs other than atheist and were thus excluded listwise. It is unknown whether these non-atheists were the same individuals who indicated “atheist” on the original pre-screener and subsequently changed their beliefs, or perhaps different individuals using those earlier atheist users’ accounts.

Data was analyzed for the remaining 222 atheists (121 women, 101 men), ranging in age from 18–80 (*M* = 41.59, SD = 37.43), with an average 14.87 years of education (SD = 3.11). Participants were mostly white (203 Caucasian, 9 African American, 3 Asian/Pacific Islander, 2 American Indian/Native Alaskan, 5 “other”) and non-Hispanic/Latino (9 Hispanic/Latino, 212 non-Hispanic/non-Latino).

### Materials and Procedure

Participants completed materials for a study described as investigating ‘‘personality and social attitudes’’ *via* a link to an online service (^1^ Provo, UT, United States) provided to them by the Cloud Research platform. After obtaining informed consent, participants completed the study materials for our 2 (MS, dental pain control) × 2 (Atheists are common, atheists are rare) factorial design on the dependent variable of meaning in life (Krause, 2004). All measures, manipulations, and exclusions for this study are reported.

#### Personal Need for Structure

To begin, participants completed a short version of the Personal Need for Structure Scale (PNS: Thompson et al., 2001). A person who scores high in PNS prefers order and certainty, and dislikes ambiguity. The scale consists of 6 items measured on 6-point Likert-type scale (1 = strongly disagree, 6 = strongly agree). Example items are: “I enjoy having a clear and structured mode of life” and “I become uncomfortable when the rules in a situation are not clear.” Prior TMT research has found that individuals low in PNS report lower levels of meaning in life following reminders of death then those high in PNS (Vess et al., 2009). In an earlier study, Vail and Soenke (2018) found that atheists in their samples had lower levels of PNS than Christian participants. The PNS Scale was included at the beginning of this study to investigate this potential individual difference variable and support the cover story, that this is research on personality.

#### Mortality Salience

Participants were randomly assigned to complete either the Fear of Death Questionnaire (Florian and Kravetz, 1983) as an MS prime or an identical scale about dental pain as a control topic prime. The questionnaires were not scored, because the questionnaire topics were experimentally manipulated to prime participants’ awareness of death vs. dental pain. The MS version of the questionnaire included questions like “I am very much afraid to die.” and “The thought of death never bothers me.” Each version used a 10 point Likert-type scale ranging from 1 *(Strongly Disagree)* to 10 *(Strongly Agree)* (Florian and Kravetz, 1983). Although the most widely used MS prime uses two open-ended questions asking participants to think about their own death (Rosenblatt et al., 1989), fear of death scales have also been widely used in TMT research as a MS prime (see Burke et al., 2010 for review). In our experience conducting fully online experiments, fewer participants respond to the open-ended primes, making it difficult to know whether they have attended to the prime and leading to loss of participant data. By using a questionnaire that participants respond to on a Likert-type scale, we feel confident that participants have thought about the questions as they respond and are primed with death.

#### Prevalence of Atheism

Following MS, participants were presented with a short passage to read about worldwide atheism rates and told that they would be asked questions about the passage later in the study to encourage them to pay attention to what they read. These passages were taken directly from Gervais (2011) to give participants some information about whether there is normative support for atheism. Half of the participants were randomly assigned to read a passage indicating that atheists are common and outnumber other established religious groups. For example, an excerpt from the passage states: “Globally, atheists are 58 times more numerous than Mormons, 41 times more numerous than Jews, and twice as numerous as Buddhists; non-believers constitute the fourth largest religious group in the world, trailing only Christians, Muslims, and Hindus (Zuckerman, 2007).” The other half of participants read a passage indicating that atheists are rare and becoming even less common worldwide (Gervais, 2011). An excerpt from this passage states: “Compared to the great world religions, atheists are fairly rare, and do not have a particularly visible worldwide presence. And, according to data from Norris and Inglehart (2004), atheists are becoming less common worldwide, relative to other religious groups.”

#### Delay and Distraction

As already noted, a large body of research indicates that the distal effects of explicit MS primes (e.g., such as the explicit questionnaire-based MS manipulation used in the present study) emerge most strongly after a delay, when death thought is highly accessible to consciousness but no longer in focal attention (Greenberg et al., 1997; Kosloff et al., 2018). To achieve this delay, participants completed the 60 item Positive and Negative Affect Scale (PANAS-X; Watson and Clark, 1994) and a brief 3–5 min reading task (Pyszczynski et al., 1999).

#### Meaning in Life

Following the delay, participants completed the full 23-item Meaning in Life Questionnaire (Krause, 2004) as the dependent measure. Participants indicated their agreement with items using a six-point Likert-type scale ranging from 1 (Strongly Disagree) to 6 (Strongly Agree). Sample items include “*I have a sense of direction and purpose in life*.” and “*I feel good when I think about what I have accomplished in life*” (Krause, 2004).

#### Demographics

At the end of the study, participants completed a demographic questionnaire assessing age, sex, race/ethnicity, education level, political orientation, and religious belief/unbelief. Participants also completed a questionnaire about response environment and a question to determine any suspicion during participation.

## Results

A 2 (MS, control) × 2 (atheists are common, atheists are rare) ANOVA was conducted on participants’ average scores for the Meaning in Life Questionnaire. Although this analysis revealed no main effect for MS [*F*(1, 218) = 1.91, *p* = 0.161, η_*p*_^2^ = 0.009] or atheists are common/rare article condition [*F*(1, 218) = 0.18, *p* = 0.676, η_*p*_^2^ = 0.001], a significant interaction emerged [*F*(1, 218) = 6.12, *p* = 0.014, η_*p*_^2^ = 0.027].

Pairwise comparisons revealed that, among participants who read that atheists are common, meaning in life was lower in the MS than in the dental pain condition [*t*(218) = --2.70, *p* = 0.007, *d* = --0.44 (95% CI: --0.83, --0.07)]. Among those who read that atheists are rare, meaning in life did not differ between the MS and pain conditions [*t*(218) = 0.76, *p* = 0.46, *d* = 0.09 (95% CI: --0.28, 0.46)].^2^ Further, among those in the MS condition, those who read that atheists are common had significantly lower levels of meaning in life than those who read that atheists are rare [*t*(218) = –2.01, *p* = 0.045, *d* = –0.43 (95% CI: –0.79, –0.03)]. Among participants in the dental pain condition, meaning in life did not significantly differ between the common and the rare condition [*t*(218) = 1.47, *p* = 0.14, *d* = 0.22 (95% CI: –0.18, 0.56)] (Table 1).

**TABLE 1:** Descriptive statistics of atheists’ meaning in life in each cell.

	Atheism common			Atheism rare		
	*M*	SD	*n*	*M*	SD	*n*
Mortality salience	3.34	0.82	52	3.64	0.63	56
Pain salience	3.73	0.89	56	3.58	0.67	58

## Discussion

The present study explored whether death awareness would or would not continue to undermine atheists’ meaning in life after exposure to normative support for atheism itself. Results indicated that MS (vs. pain salience) reduced atheists’ perceived meaning in life when atheists were informed that atheism is common but not when informed atheism is rare.

This data pattern did not support the worldview affirmation hypothesis, which was built on (1) the assumption that normative support for atheism would affirm atheists’ worldview beliefs, standards, and values, and (2) the TMT idea that affirming one’s permanence-promising worldviews would help manage existential concerns about one’s impermanence. As mentioned in the section “Introduction”, much prior research is consistent with the latter idea—demonstrating that social support and consensus can play an important role in buffering against existential threat (Greenberg et al., 2014). Thus, the present data suggest the former assumption was incorrect—that ostensible normative support for atheism does *not* affirm any particular secular permanence-promising worldviews.

Rather, the observed data pattern was consistent with the worldview negation hypothesis, which recognized that atheism does not describe the presence of any particular secular terror-managing worldview beliefs but merely the absence or rejection of religious terror-managing beliefs. From that view, a prime suggesting atheism is rare, such that traditional religious worldviews are still prevalent, would not have negated the long-standing and familiar sociocultural systems within which atheists have always trafficked. Even if one is atheist, one can still know and meaningfully navigate toward a permanence-promising legacy within the Western Judeo-Christian sociocultural landscape even while ignoring or rejecting the supernatural concepts associated with it. In contrast, a prime suggesting growing global consensus for atheism would perhaps represent a broader negation of such religious worldviews—but without necessarily affirming any particular secular worldview in its place. That groundlessness might have left atheist participants vulnerable to reduced meaning in life when reminded of mortality, which fits data patterns observed in the present study. These findings could be seen as consistent with an incident in Don Delillo’s novel about the fear of death, *White Noise* (Delillo, 1985); in the novel, an atheist character becomes disturbed when he learns that a nun also disbelieves in a supernatural higher power. It may be that non-believers get some comfort or value from other people being believers.

### Implications, Considerations, and Future Directions

The present data patterns raise several considerations that are worth considering further, including some with implications that may spur informative future research.

#### Compensation Hypothesis

The first stems directly from the present findings and considers how atheists might maintain meaning in life and manage existential concerns. The idea we propose here is that, to maintain existential wellbeing, the atheist absence or rejection of a meaningful terror-managing religious faith must be compensated for by the presence of a meaningful terror-managing set of secular worldview beliefs, standards, and values. Others have similarly proposed that science can, at least in part, compensate for religion and serve an analogous psychological function (Farias et al., 2013). Indeed, MS increased support for evolutionary theory among natural science students (Tracey et al., 2011), and among atheists the typical MS-induced secular worldview defense was eliminated after a prime affirming life extension based on medical science (Vail et al., 2020).

But secular compensation requires not just scientific information and technological advances, it also entails moral, social, and cultural engagement. It means learning non-supernatural explanations about the world (science), developing a sense of purpose through secular (perhaps humanist) social standards and moral values and goals, and adopting or developing meaningful cultural paths toward living up to those worldviews. Thus, an existentially healthy individual might indeed be an atheist, but one who not only (a) lacks or rejects religious faith, but also (b) compensates for that through scientific learning and knowledge, the adoption of guiding secular humanist values (e.g., education, compassion), and a sense of purpose and legacy through secular participation in a larger and longer-lasting cultural system—arts, sports, family, civic engagement, and so on.

In that light, the present research found the “atheism is common” prime did not buoy atheists’ meaning in life when reminded of death, which would be expected because the prime did not also compensate for that negation of religion by offering in its place an affirmation of participants’ secular beliefs, standards, or values. Thus, future research could test whether an affirmation of atheists’ secular worldviews would help them maintain perceived meaning in life after being reminded of death.

#### Normative Support Prime: A Backfire Effect?

One possible alternative explanation for the present results might argue that the worldview affirmation hypothesis is still true, and that a compelling and pervasive social support for atheism would serve a buffering function for atheists, but that the “atheism is common” prime in the present study backfired. The sample of atheists recruited in this study were American atheists. Although the “religious nones” in the United States are a growing minority, atheism remains just a small minority at 3% of the population—an estimated 9.62 million of the 2015 population of 320.7 million Americans (Pew Research Center, 2015a). Further, anti-atheist prejudice is prevalent and strong (Edgell et al., 2006; Gervais et al., 2011; Jones, 2012) so many atheists remain “closeted” (Gervais and Najle, 2018), hiding their unbelief from others. The result is that most atheists are unlikely to encounter other atheists, or even hear about support for atheism, in their daily lives. Thus, it is possible that, rather than increasing social support and validation, the present “atheists are common” prime may have actually engendered a sense of isolation by drawing attention to the many atheists out in the rest of the world while one is isolated in their atheism here in the United States.

This alternative perspective can potentially explain why MS still reduced atheists’ meaning in life in the “atheism is common” prime condition. However, it would have a difficult time explaining why MS had no effect in the “atheism is rare” condition. If being inadvertently reminded of one’s atheist isolation makes one vulnerable to reduced meaning in life after existential threat, then surely an overt and direct reminder would produce the same effect—and yet it did not. Perhaps learning that atheists are rare may validate their sense of isolation and even help to explain it, and thereby somehow buffer against existential threat to well-being. But, it is difficult to see why the “atheism is rare” prime would not, for the very same reasons, undermine social support for atheism as a potential death-denying worldview system—which should have created a vulnerability to reduced meaning in life after a death reminder. Thus, the “backfire effect” (isolation) idea encounters problems explaining the full data pattern.

#### Atheism as an Achievement: A Source of (Self-)esteem?

Another alternative builds on the possibility that American atheists might feel some terror-managing sense of uniqueness, achievement, or even superiority from being one of the few atheists in America. The underlying assumption here being that, in a nation saturated with irrational and factually unverifiable religious concepts, the American atheist may interpret having become atheist as a rare and commendable intellectual achievement! Indeed, research finds unbelief is correlated with higher intelligence (Zuckerman et al., 2013), higher levels of education (Strieb and Klein, 2013), greater analytic reasoning (Pennycook et al., 2012; Shenhav et al., 2012), and an emphasis on scientific thinking (Larson and Witham, 1998; McCauley, 2011). Research on anti-theists specifically has shown that they see the rejection of religion as overcoming immature thought to reach a higher insight (Bulbulia, 2005).

Thus, if American atheists regard their atheism as an uncommon intellectual achievement, then it’s possible the “atheism is rare” prime could have bolstered their self-esteem and buoyed their meaning in life after MS, whereas the “atheism is common” prime would have threatened the uniqueness of that achievement and left them vulnerable to reduced meaning in life when reminded of death. The present data patterns seem to fit with this idea, though we suggest some caveats. First, some skepticism may be warranted, as our admittedly anecdotal experience suggests very few atheists regard their atheism as a source of pride—rather, it’s often a struggle. Second, atheism is hardly a source of social status/esteem in the normative American sociocultural landscape; as described above, recent research shows atheism is still highly stigmatized and the target of strong prejudice by the religious. So, it seems unlikely that the relatively rare “achievement” of atheism in America would be taken as a source of social esteem, but it might be a more limited and private source of positive *self*-regard. Third, if so, such would likely only be the case for the subset of anti-theists (Silver et al., 2014)^3^ from the subset of atheists who explicitly recognize their own atheism (e.g., Flew, 1984; Martin, 1992). Thus, while possible, there are important caveats and limiting considerations that warrant skepticism.

### Limitations

The generalization of the present data is of course limited to the American context. The prevalence of atheism and the corresponding importance of secular, rather than religious, worldviews varies dramatically across nations and cultural regions (Keysar and Kosmin, 2007; Streib et al., 2009). Our sample of American atheists should be understood in the broader context of the American religious landscape, where there are fewer atheists, more anti-atheist prejudice, and aspects of religion, particularly Judeo-Christian values, are embedded into the fabric of society. Cross-cultural research would be needed to learn whether this finding is isolated to American atheists or whether it generalizes to atheists residing in countries where atheism is more prevalent and accepted, such as Scandinavia or New Zealand.

A related limitation results from our reliance on one single question to determine our atheist sample. Some theorists and researchers have suggested that there are different kinds of atheists (Norenzayan and Gervais, 2013; Holmes et al., 2021). For example, for some, atheism may be a central aspect of their worldview, and something that they invest in and want to spread to others. For others, their lack of belief may just reflect a lack of exposure to deistic beliefs or a lack of cognitive orientation or intuition regarding supernatural agents. Some atheists may view the world optimistically, embracing secular humanism, whereas others may embrace a more nihilistic, absurdist worldview. If we had differentiated between different types of atheists based on how they integrate their disbelief within their worldview or bases of self-worth, we might have found that for some atheists, the combination of MS and “atheists are common” would undermine meaning in life, as our results suggest, but for others, it would not. This may be a fruitful avenue for further research on how MS and the prevalence of atheism relate to meaning in life.

Additionally, the present work is limited to the outcome of perceived meaning in life, but future directions for this research could explore additional outcome measures like physical health or affective well-being (e.g., happiness). There is research to suggest that meaning in life may play a different role for atheists than it does for religious individuals. The literature on meaning in life indicates that for religious individuals, meaning in life is highly related to both physical health and psychological well-being (see Hood et al., 2018 for a review). Steger and Frazier (2005) found that meaning in life significantly mediated the relationship between religion and personal well-being; suggesting that religion’s role in providing meaning is an important aspect of why religion is associated with positive outcomes. But for atheists, lower ratings of meaning in life do not translate to lower overall happiness or life satisfaction in the way they do for religious individuals (Horning et al., 2011; Schnell and Keenan, 2011). Given this, investigating the relationship between religiosity, meaning in life, and other positive outcomes from a TMT perspective is an important next step for empirical research into the psychology of religion.

## Conclusion

Building on prior research (Vail and Soenke, 2018), the present work explored whether death awareness would undermine atheists’ meaning in life after exposure to normative support for atheism itself. Results indicated death awareness did reduce atheists’ meaning in life when given information that atheism is common. These data show that salient prevalence of atheism does not appear to function as an affirmation of a permanence-promising worldview, consistent with the idea that atheism does not describe the presence of a particular secular worldview but rather only the absence or rejection of religious supernatural concepts.

## Data Availability Statement

The raw data supporting the conclusions of this article will be made available by the authors, without undue reservation.

## Ethics Statement

The studies involving human participants were reviewed and approved by the California State University Channel Islands IRB. The patients/participants provided their written informed consent to participate in this study.

## Author Contributions

MS and KV contributed to the conception and design of the study. JG helped with operationalizing variables. MS collected and organized the participant data and performed the statistical analysis. MS wrote the first draft of the manuscript. MS, KV, and JG wrote sections of the manuscript. All authors contributed to manuscript revision, read, and approved the submitted version.

## Conflict of Interest

The authors declare that the research was conducted in the absence of any commercial or financial relationships that could be construed as a potential conflict of interest.

## Publisher’s Note

All claims expressed in this article are solely those of the authors and do not necessarily represent those of their affiliated organizations, or those of the publisher, the editors and the reviewers. Any product that may be evaluated in this article, or claim that may be made by its manufacturer, is not guaranteed or endorsed by the publisher.

## Footnotes

1
http://qualtrics.com/

2 2 (MS, control) × 2 (atheists are common, atheists are rare) (M) ANOVAs conducted on PNS and all 13 of the PANAS-X subscales revealed only a small main effect for MS condition on fatigue *F*(1, 218) = 3.85, *p* = 0.051, η*p*^2^ = 0.017, with participants in the MS condition reporting higher fatigue (*M* = 3.62, SD = 1.21) than those in the control condition (*M* = 3.29, SD = 1.12).

3 The six types of non-belief identified by Silver et al. (2014) include: (1) ritual atheists/agnostics, who reject belief in God, but continue to participate in religious traditions for extrinsic reasons; (2) non-theists, who do not think about religion and so see it as a non-issue; (3) anti-theists, who actively seek to end religion; (4) seeker-agnostics, who feel people can’t know whether religion is correct or not; (5) activist atheists/agnostics, who are motivated by belief in secular worldviews rather than religious; and (6) intellectual atheists/agnostics, who favor scientific or philosophical arguments against religious belief.
